# Evaluating the concordance of pollen forecasting apps against automated pollen monitoring: A single-site experience

**DOI:** 10.1016/j.jacig.2026.100639

**Published:** 2026-01-12

**Authors:** Freddy Gonzalez, Christina E. Ciaccio, Sharmilee M. Nyenhuis

**Affiliations:** a University of California Irvine, Irvine, Calif; b Section of Allergy and Immunology, Department of Pediatrics, University of Chicago, Chicago, Ill

**Keywords:** Pollen, allergy, asthma, air quality, eHealth, mobile apps, environmental monitoring

## Abstract

**Background:**

Individuals with allergic rhinitis and asthma rely on accurate pollen forecasts to avoid allergen exposure and manage symptoms. However, many widely used weather and health applications (apps) use manual pollen counting methods, which may vary in accuracy.

**Objective:**

This study aimed to evaluate the concordance between popular pollen forecasting apps and real-time data collected from an automated pollen monitoring device at a single site in the Chicago area.

**Methods:**

We compared daily pollen forecasts from 2 commonly used consumer apps (The Weather Channel app and the AccuWeather app) with pollen data recorded by the PollenSense automated monitoring device over 2 months. To assess daily concordance, forecasted pollen levels and automated counts were categorized as being in the low, moderate, or high ranges. Descriptive and inferential assessment of accuracy and reliability of consumer-facing pollen forecasts were performed.

**Results:**

Across the study period, concordance between the consumer apps and the PollenSense counts was low (the forecast levels for the AccuWeather app were 7% for grass, 33% for ragweed, and 56% for mold, whereas those for The Weather Channel app were 29% for grass and 34.% for ragweed). No statistically significant association was found between the pollen forecasts and measured pollen levels.

**Conclusion:**

The popular pollen forecasting apps demonstrated poor concordance with real-time automated pollen data. These findings highlight the limitations of current forecasting tools and underscore the need for improved, validated technologies to support clinical decision making and public health recommendations for individuals affected by pollen allergies.

## Introduction

Outdoor aeroallergens, including tree, grass, and weed pollens as well as mold spores, are well-established triggers for allergic rhinitis and asthma. Trigger avoidance is a cornerstone of disease management and often includes monitoring pollen levels through consumer-facing weather and allergy forecast applications (apps). However, the accuracy of these apps—particularly in comparison with that of real-time, automated pollen monitoring systems—remains underexplored. This study sought to evaluate the agreement between pollen level forecasts from 2 popular consumer apps (The Weather Channel App and the AccuWeather app) and data from an automated pollen counter (PollenSense) in Chicago, Illinois.

## Results and discussion

**Fig 1:**
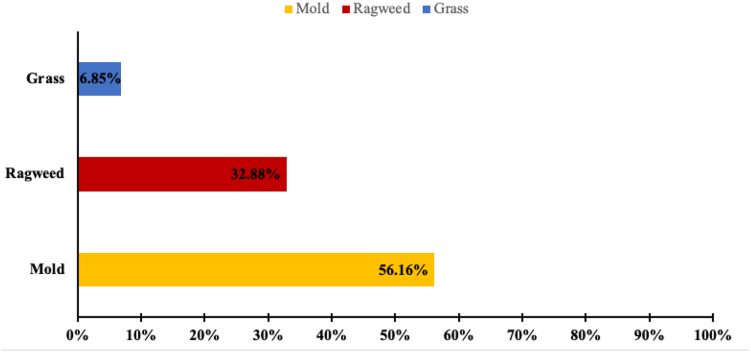
Concordance between the AccuWeather app and PollenSense.

**Fig 2:**
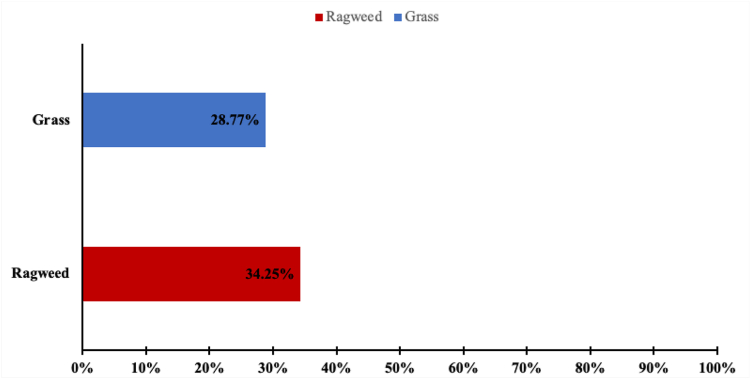
Concordance between The Weather Channel app and PollenSense. The Weather Channel lacks data on mold.

Overall, the concordance between the popular consumer apps and the automated pollen counter (PollenSense) was low. The AccuWeather app showed the highest concordance with the automated pollen counter (specifically, about half [56%] of the time for mold), but for ragweed it was concordant only a third (33%) of the time (Fig 1). The Weather Channel app demonstrated lower agreement, with 34% concordance for ragweed and 29% for grass (Fig 2). Both consumer apps reported tree pollen data daily throughout the study period, whereas PollenSense reported no tree pollen for any day during the same timeframe, resulting in 0% concordance for this pollen type. To further strengthen the evaluation of pollen forecasting apps, diagnostic accuracy metrics, including sensitivity, specificity, positive predictive value, negative predictive value (NPV), and F1 scores (see Table E1 available in the Online Repository at www.jaci-global.org) were calculated. The AccuWeather app demonstrated stronger predictive accuracy for ragweed (precision, 0.702; recall, 0.767; and F1 score = 0.733) than The Weather Channel (precision, 0.686; recall, 0.545; and F1 score = 0.608). However, both apps performed poorly in terms of NPV and specificity (≤0.39), reflecting limited ability to correctly identify true low-pollen days. In contrast, for grass pollen, the apps performed comparably, with low precision and recall (F1 score = 0.342 for both) but higher NPV and specificity (>0.64), reflecting better performance at ruling out grass pollen events than detecting them.

The AccuWeather app and The Weather Channel presented their data in categoric formats (low, moderate, high, and very high), whereas PollenSense provided numeric values. To ensure comparability, the numeric data were converted into the appropriate categories using Table E2 (available in the Online Repository at www.jaci-global.org). To assess whether forecast categories and actual pollen levels were statistically associated, the Fisher exact test was used. No statistically significant association was found for any of the 4 comparisons with odds ratios approaching 1, indicating weak or negligible association between forecast categories and actual pollen levels (see Table E3 available in the Online Repository at www.jaci-global.org). The findings did not change when the popular app data were adjusted by 1 day, as the pollen forecasts may lag 1 day behind those at PollenSense.

Our analysis demonstrated that diagnostic accuracy varied considerably across consumer apps, with overall concordance between forecasts and observed pollen levels being low. This highlights the tendency toward false-positive results, particularly for tree and ragweed forecasts. The presence of false positives in the consumer apps is concerning and may carry negative clinical implications, as it may lead to unnecessary alterations in daily activities, which is not ideal for effective allergy management. The incorporation of diagnostic metrics into our analysis not only clarifies the strengths and weaknesses of each consumer app but also underscores their clinical relevance: high sensitivity reduces missed exposures, whereas higher specificity and positive predictive value are critical to minimize unnecessary lifestyle disruptions. To more accurately characterize and compare the reliability of digital forecasting tools, future studies should continue to adopt this framework.

Previous work has shown findings similar to those of our work, with concordance rates less than 60% even when allowing tolerances of 4 pollen grains/m^3^.^1^ The discrepancies may arise from differences in data infrastructures, as many apps depend on regional predictive models rather than direct automated pollen quantification. This is particularly relevant to our study, as it highlights the broader issue of limited forecast accuracy across commonly used apps. By directly comparing forecasted categories with actual pollen counts, our analysis builds on these prior findings and provides a more detailed evaluation of diagnostic accuracy.

Few studies have directly compared consumer-facing apps with real-time, site-specific automated counters. The discrepancies observed in our study may stem from differences in sampling methods—such as regional averaging used by apps versus hyperlocal, real-time monitoring used by automated counters—as well as differences in thresholds for pollen categorization and potential lag times in data updates. Although emerging technologies such as low-cost particle sensors and machine learning models show promise for scalable monitoring, their implementation requires rigorous benchmarking against criterion standard counter methods such as Hirst-type traps.^2^ Clinicians should advocate for transparency initiatives in consumer

pollen apps while directing high-risk patients toward validated, real-time monitoring platforms until regulatory standards for allergy-focused eHealth tools are established.^3^

Our study focused exclusively on forecast concordance but did not have access to any reported symptoms. Nonetheless, recent research has begun to address this gap by examining how pollen forecasts can directly influence allergy symptoms and patient outcomes. One randomized controlled trial demonstrated that a multifunctional allergy app, particularly one with a pollen forecast feature, significantly improved symptom management and quality of life for individuals with grass pollen allergy.^4^ Participants using the full-featured app reported fewer symptoms, increased medication adherence, and reduced social activity impairment than did those using limited-function versions. These findings validate the potential clinical value of accurate pollen forecasts, as improving forecast reliability may further enhance symptom control and quality of life for individuals with allergy.

Recent research has demonstrated the potential of leveraging crowdsourced symptom data to improve pollen forecasts.^5^ The concept that apps could "learn" from user-reported symptoms to adjust predictions in real time is promising. Such next-generation eHealth tools have the potential to provide highly personalized and context-specific allergy forecasts, improving both accuracy and clinical utility. Expanding on this vision, future systems could integrate environmental monitoring with longitudinal symptom tracking, data from wearable sensors, and medication use patterns to generate adaptive, individualized risk alerts. Incorporating machine learning algorithms would allow apps to continuously refine predictions at both the individual and population levels, addressing the lack of agreement across pollen categories seen in our study. Ultimately, these tools could move beyond static forecasts to become dynamic platforms for precision allergy management, offering targeted interventions that better support daily decision making for individuals with allergy. Future research should focus on developing standardized quality control frameworks for pollen forecasts and exploring how real-time, high-resolution data can be effectively communicated to patients and health care providers.

A limitation of this study is potential exposure misclassification, as individuals’ actual allergen exposure may differ from local measurements owing to daily mobility. A possible solution is to incorporate Global Positioning System–based mobility data in future research that could improve the accuracy of exposure assessments. Additionally, this study relied on categoric concordance, whereas future work incorporating continuous analyses may offer a more nuanced understanding of forecast concordance.

In closing, this study reveals poor concordance between pollen level forecasts provided by widely used consumer apps and those generated by an automated, real-time pollen counter. For patients with asthma or allergic rhinitis who depend on these tools for exposure avoidance, the use of inaccurate data may hinder effective symptom management. Without transparency about the underlying data and predictive algorithms, the scientific validity of pollen forecasts remains unverifiable, undermining both public trust and clinical applicability. These findings emphasize the need for more robust validation of pollen forecasting tools, and they encourage the integration of real-time automated monitoring into consumer health apps.

For detailed methods, please see the Methods section in this article's Online Repository at www.jaci-global.org.

**Clinical implications**

Accurate pollen data are vital for allergy management. This study reveals poor concordance between popular consumer apps and automated pollen monitors, emphasizing the need for validated pollen forecasting tools in clinical practice.

## Disclosure statement

Disclosure of potential conflict of interest: S. M. Nyenhuis receives funding from the National Institutes of Health (NIH) and the Allergy and Asthma Foundation of America, has served on an advisory board for GSK, and receives royalties from Wolters-Kluwer and Springer. C. E. Ciaccio receives funding from the NIH, Genentech, and Food Allergy Research and Education and has served as an adviser for Siolta, Clostrabio, Novartis, and Sanofi. The remaining author declares no relevant conflicts of interest.

## Supplementary data

Supplementary Methods

Supplementary Tables E1 and E3
