# Supplementary material for: Evaluating the concordance of pollen forecasting apps against automated pollen monitoring: A single-site experience
Source: J Allergy Clin Immunol Glob. 2026 Jan 12;5(2):100639. doi: 10.1016/j.jacig.2026.100639 (PMC12834900; doi:10.1016/j.jacig.2026.100639)
Supplement: Supplementary Methods [file mmc1.docx]

| **Pollen Type** | **App** | **Precision (+ PPV)** | **Recall (Sensitivity)** | **F1-Score** | **NPV** | **Specificity** |
| --- | --- | --- | --- | --- | --- | --- |
| Ragweed | Weather Channel | 0.686 | 0.545 | 0.608 | 0.259 | 0.389 |
| Ragweed | AccuWeather | 0.702 | 0.767 | 0.733 | 0.286 | 0.222 |
| Grass | Weather Channel | 0.318 | 0.368 | 0.342 | 0.692 | 0.643 |
| Grass | AccuWeather | 0.318 | 0.368 | 0.342 | 0.7 | 0.651 |

**Table E1. Diagnostic performance of consumer apps for ragweed and grass pollen**

**Table E2. National Allergy Bureau categorization of pollen levels for each pollen type**

| **Pollen Types** | | | | |
| --- | --- | --- | --- | --- |
|  | **Tree** | **Weed** | **Grass** | **Mold** |
| Low | 1 to 14 ppm^3^ | 1 to 9 ppm | 1 to 4 ppm^3^ | 1 to 6,499 ppm^3^ |
| Moderate | 15 to 89 ppm^3^ | 10 to 49 ppm^3^ | 5 to 19 ppm^3^ | 6,500 to 12,999 ppm^3^ |
| High | 90 to 1,499 ppm^3^ | 50 to 499 ppm^3^ | 20 to 199 ppm^3^ | 13,000 to 49,999 ppm^3^ |
| Very High | More than 1,500 ppm^3^ | More than 500 ppm^3^ | More than 200 ppm^3^ | More than 50,000 ppm^3^ |

**Table E3. Association between forecasted and observed pollen levels for ragweed and grass pollen across both consumer apps**

| **Pollen Type** | **App** | **Odds Ratio** | **P-Value** |
| --- | --- | --- | --- |
| Ragweed | Weather Channel | 0.764 | 0.78 |
| Ragweed | AccuWeather | 0.943 | 1 |
| Grass | Weather Channel | 0.969 | 1 |
| Grass | AccuWeather | 1.089 | 1 |
