# Supplementary material for: Evaluating the concordance of pollen forecasting apps against automated pollen monitoring: A single-site experience
Source: J Allergy Clin Immunol Glob. 2026 Jan 12;5(2):100639. doi: 10.1016/j.jacig.2026.100639 (PMC12834900; doi:10.1016/j.jacig.2026.100639)
Supplement: Supplementary Tables E1 and E3 [file mmc2.docx]

**Online Repository Methods**

Daily pollen level data were collected from July 8, 2024, to September 19, 2024, for grass, weed (specifically ragweed), and mold allergens. Data was extracted into an excel sheet from two widely used consumer apps—AccuWeather and The Weather Channel—and from PollenSense, an automated pollen counter.

Each pollen type was categorized using ordinal categories provided by each app: low, moderate, high, or very high (Table E2). The National Allergy Bureau (NAB) table is the standard in the U.S. for classifying pollen and mold levels into categories such as low, moderate, high, and very high, based on the measured counts (grains or spores per cubic meter of air) over a 24-hour period. Concordance was defined as the percentage of days where the consumer app and PollenSense data assigned the same category for a given pollen type. As the data from PollenSense was from an automated pollen counter, this was used as the reference. All data were collected from the same ZIP code to ensure location consistency.

To evaluate forecast performance, we applied diagnostic accuracy metrics, including sensitivity, specificity, positive predictive value (PPV), and F1 scores. These measures allowed us to assess each app’s ability to correctly identify days with elevated pollen versus low pollen exposure. Initially, we aimed to apply chi-square tests; however, some cells in the contingency tables contained zeros, which violates chi-square assumptions. To address this, we collapsed the original scale into two levels: “low” and “elevated” and used Fisher’s exact test. This approach enabled us to construct 2×2 contingency tables and formally test the statistical association between app forecasts and observed pollen levels. Fisher’s exact test results provided odds ratios and p-values for each pollen type and platform. Together, these analyses offered both descriptive and inferential insight into the accuracy and reliability of consumer-facing pollen forecasts.
